# Management Strategies, Challenges, and Clinico-Radiological Outcomes of Floating Knee Injuries in Jharkhand: A Prospective Study

**DOI:** 10.7759/cureus.75660

**Published:** 2024-12-13

**Authors:** Govind K Gupta, Sabyasachi Kundu, Vinay Prabhat, Verma Dipak K Prakash Prasad

**Affiliations:** 1 Orthopedics, Rajendra Institute of Medical Sciences, Ranchi, IND; 2 Trauma and Orthopedics, Rajendra Institute of Medical Sciences, Ranchi, IND

**Keywords:** floating knee, fracture, fraser classification, karlstrom criteria, prospective study

## Abstract

Background: Floating knee injuries, involving simultaneous fractures of the femur and tibia in the same limb, present complex challenges in management. These injuries are often associated with high-energy trauma and carry significant morbidity.

Aims and objectives: This study aims to evaluate the management strategies, challenges, and clinico-radiological outcomes of floating knee injuries in Jharkhand.

Methods and materials: A prospective study was conducted involving patients with floating knee injuries treated at a tertiary care center in Jharkhand. Patients were classified based on the Fraser classification system. Treatment modalities included surgical approaches, tailored to the specific needs of each patient. Data on demographics, injury mechanisms, treatment methods, complications, and outcomes were collected and analyzed. Radiological assessments of fracture union were performed at nine, 12, and 18 months post-injury. The study included patients, with ages between 18 and 70 years. At 18 months follow-up, radiological union rates for femur and tibia fractures were assessed. Functional outcomes were assessed using the Karlstrom criteria at 18 months.

Results and conclusions: Floating knee injuries in Jharkhand predominantly result from high-energy trauma, with road traffic accidents being the leading cause. Effective management requires a multidisciplinary approach, addressing both clinical and socioeconomic challenges. A significant association has been observed between open and closed injury with both radiological and functional outcomes. The study highlights the need for improved healthcare infrastructure and patient education to enhance outcomes.

## Introduction

Ipsilateral fractures of the tibia and femur, also known as the floating knee, a term coined by Blake and McBryde in 1975, are rare injuries that typically occur in polytrauma patients. These fractures are high-energy injuries and are often associated with other severe and potentially life-threatening injuries [1].

In 1978, Fraser et al. performed a prognostic classification of the floating knee. Type I fractures include diaphyseal fractures of the femur and tibia without involving the knee joint. Type II involves fractures extending into the knee joint which are further divided into three subtypes: type IIa includes fractures involving the tibial plateau, type IIb involves distal femur fractures with knee involvement, and type IIc includes fractures involving both the tibia plateau and distal femur with the knee joint [2].

The force necessary to fracture the femur and tibia, which are considered the strongest bones in the body, is enormous. The injury mechanism is generally high-energy trauma, very often leading to marked damage to other organ systems and the affected lower limb. Intensive stabilization is required for patients with floating knee injuries having unstable hemodynamics to prevent collapsing from shock [3]. Since these injuries are caused by high-energy trauma, associated complications play a major role in deciding the timing and order of surgical intervention. In elderly patients, pre-existing medical conditions can further reduce their physiological state. There is no standard method of managing floating knee injuries. The ideal method of fracture fixation depends on many parameters such as fracture pattern, soft tissue involvement, additional injuries, and the expertise of the surgeon [4]. Floating knee injuries are most commonly seen in patients with severe trauma involving open fractures and extensive skin damage, with or without neurovascular damage. These injuries are life-threatening with mortality rates approaching up to 10% and limb amputation rates reaching up to 26% [5,6].

The initial treatment strategy is guided by the patient's overall clinical condition and the extent of local skin damage. Damage control orthopedics is the preferred approach. While surgical planning and selecting the appropriate fixation device can be complex for orthopedic surgeons, current literature advises stabilizing the femur first to simplify tibial management. This approach also permits delaying tibia fixation in patients who are medically not fit for surgery [5,7].

In this prospective study, we are going to manage and assess the clinical, functional, and radiological outcomes of all types of floating knee injuries and evaluate the challenges encountered in treating these complex injuries.

## Materials and methods

The study was a prospective longitudinal study that was conducted at the Department of Orthopedics, Rajendra Institute of Medical Sciences, Ranchi, Jharkhand, India. Approval was obtained from the Institutional Ethics Committee of Rajendra Institute of Medical Sciences (approval number: 109). The study was conducted for a duration of 1.5 years.

A total of 40 cases were included in this study. The inclusion criteria included patients aged between 18 and 70 years with floating knee injuries. In contrast, the exclusion criteria include patients aged less than 18 years and more than 70 years, patients with a previous history of any orthopedic operation of the involved or contralateral lower limb, patients with pathological fractures or any other associated ipsilateral or contralateral fracture of the lower limb, and patients having serious medical comorbidities.

**Figure 1:**
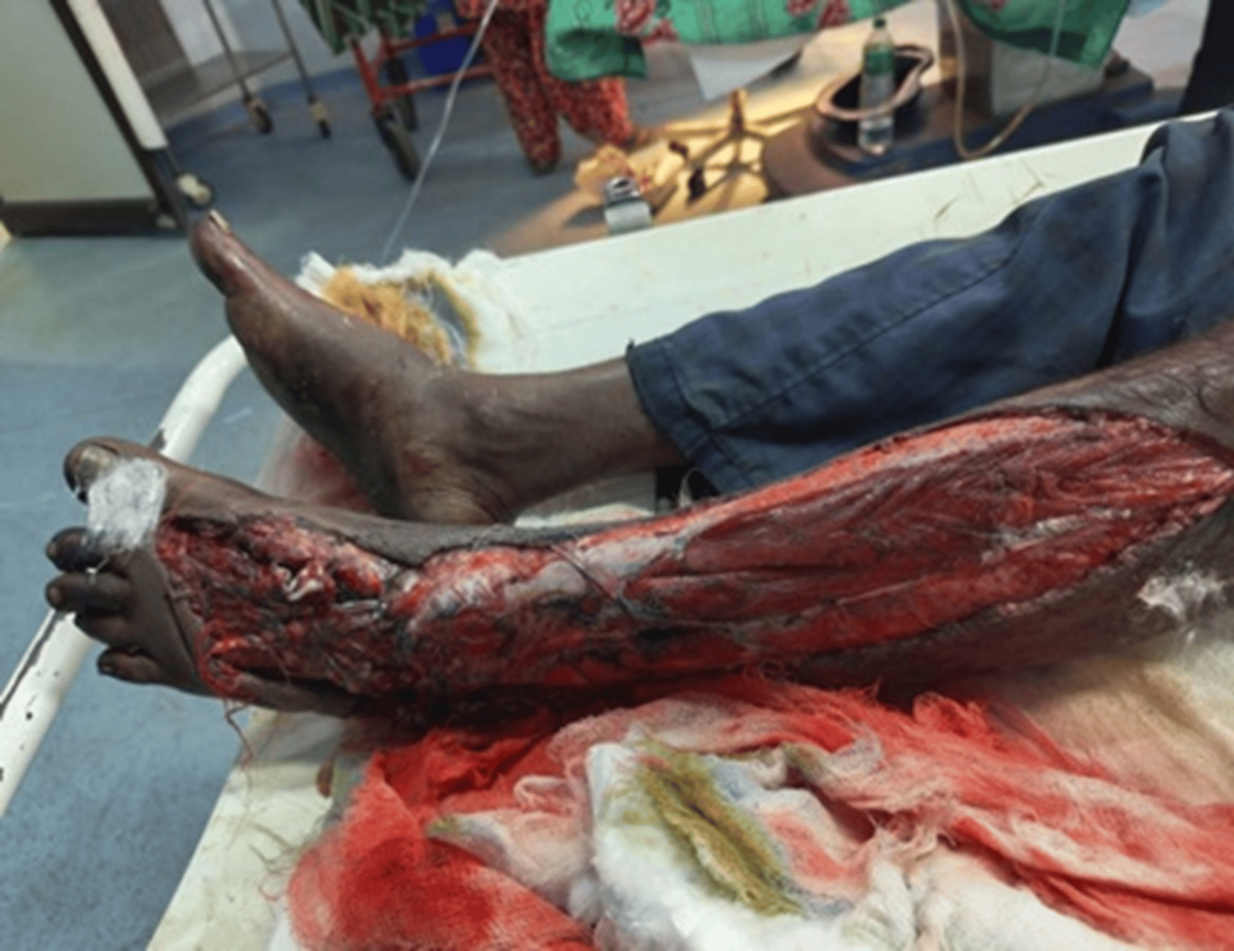
Clinical photograph showing a degloving injury of the left leg and foot with underlying fracture of the femur and tibia

**Figure 2:**
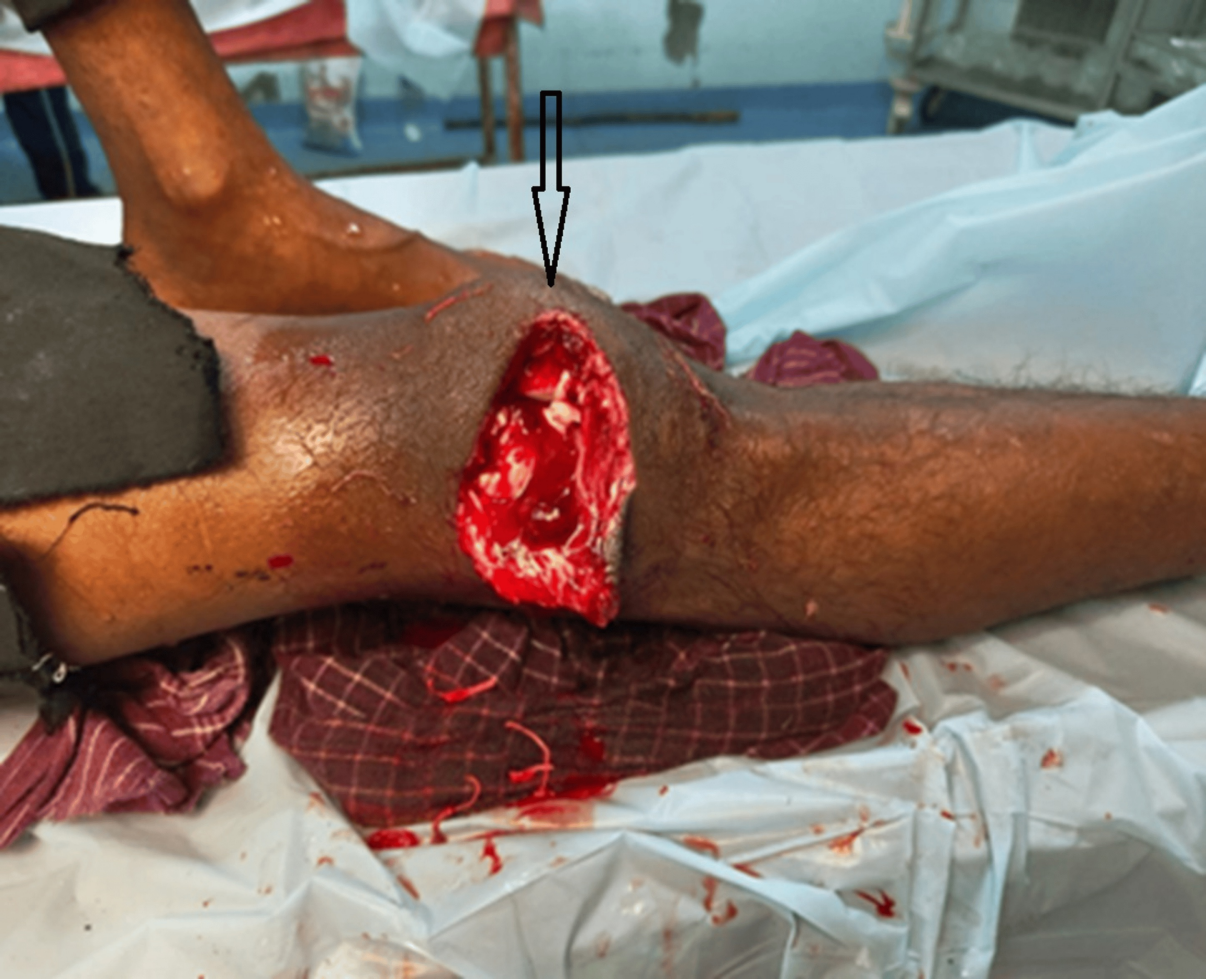
Clinical photograph showing an open fracture of the right distal femur

The study was performed in patients with ipsilateral femur and tibia fractures. The injury patterns, surgical procedures, complications, and outcomes were recorded in a pre-designed proforma. Patients were monitored for a period of 18 months and initially managed in the emergency room following Advanced Trauma Life Support (ATLS) guidelines. For open wounds, treatment included irrigation with hydrogen peroxide, normal saline, and Betadine solution, followed by the administration of appropriate antibiotics and prompt debridement (Figure 1 and Figure 2).

**Figure 3:**
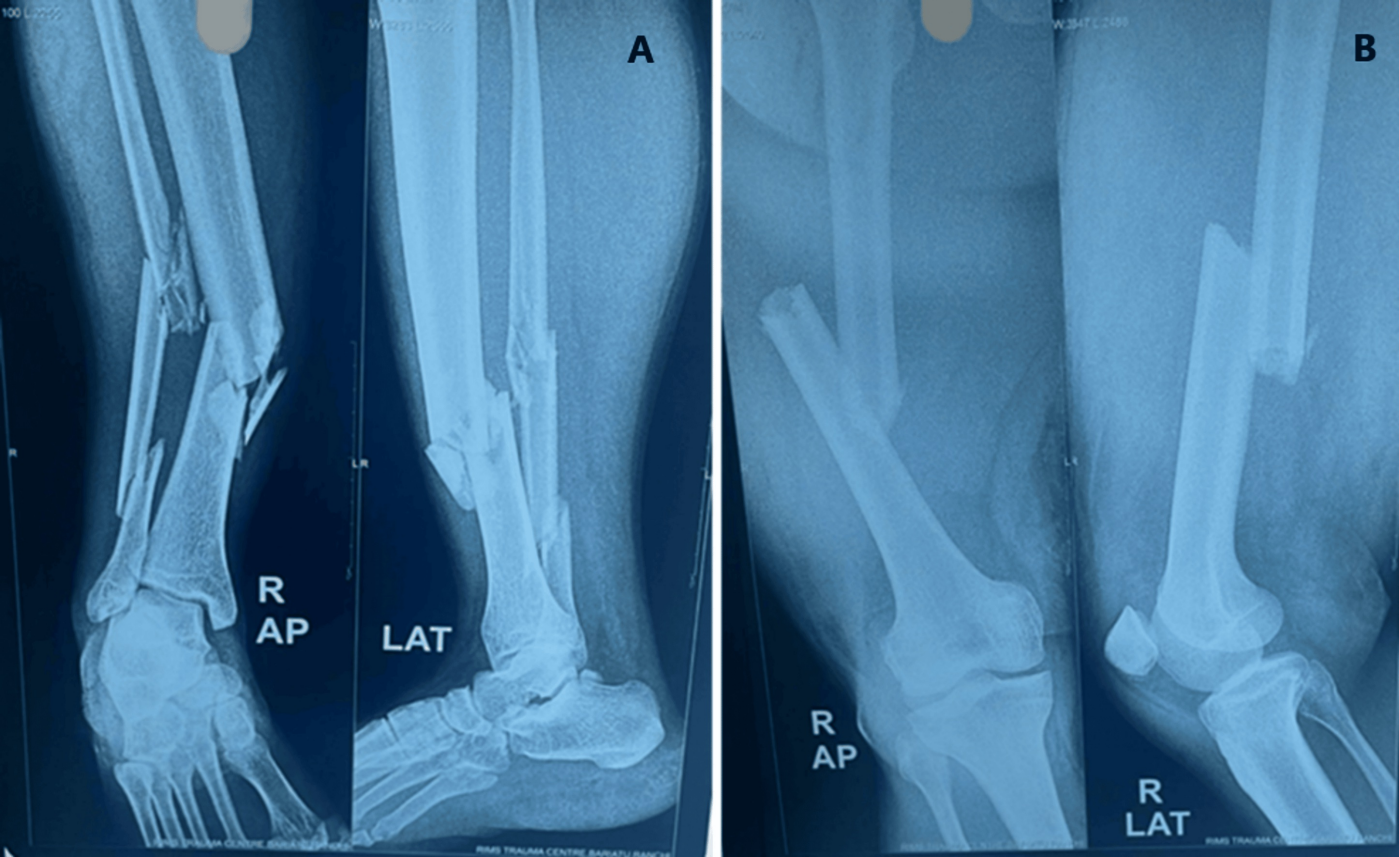
Preoperative radiograph showing a fracture of the shaft of the tibia and the femur (closed) indicating a type I floating knee injury: (A) mid-shaft of the tibia fracture and (B) mid-shaft of the femur fracture

**Figure 4:**
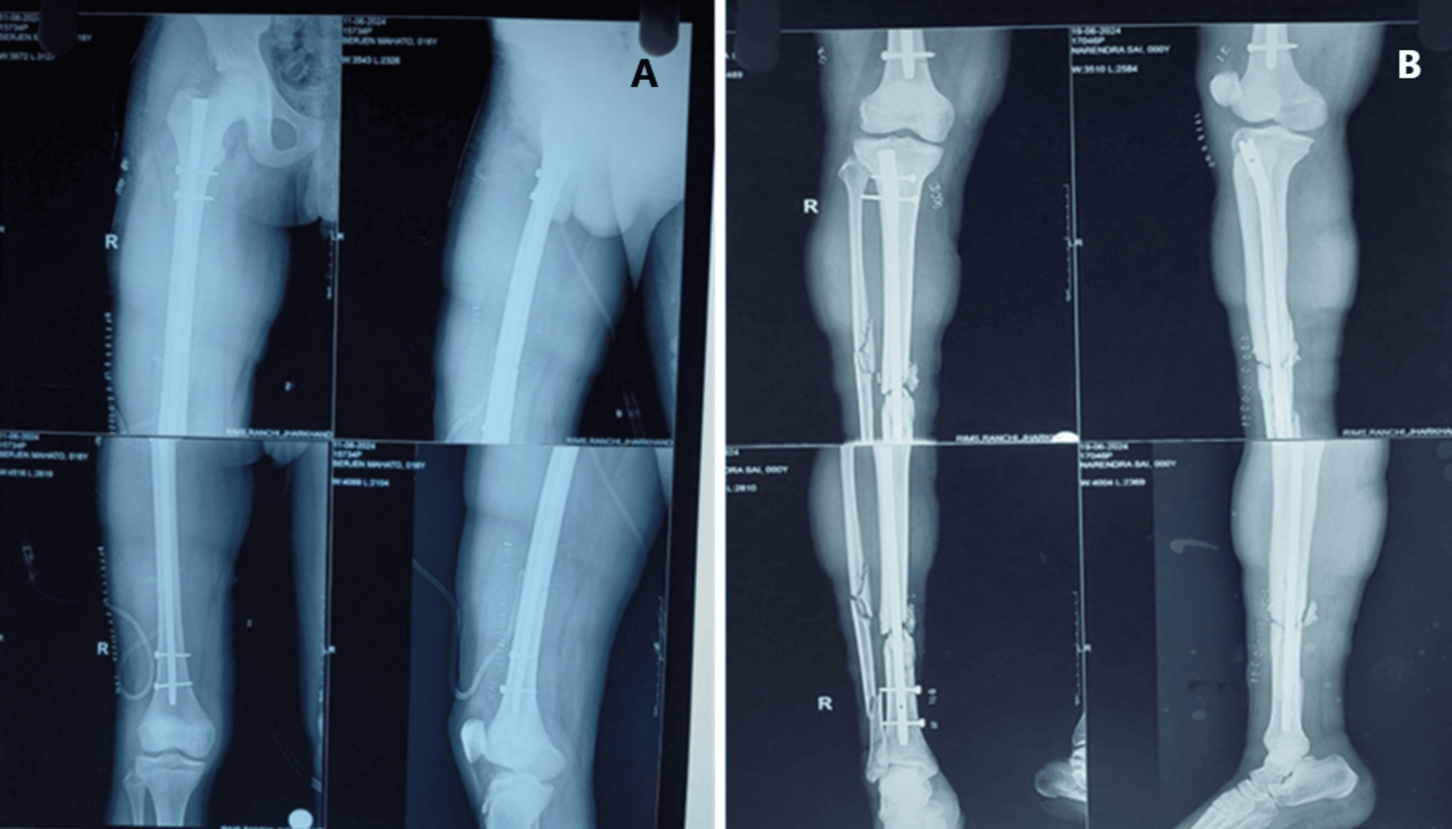
Postoperative radiograph showing internal fixation with nailing in type I floating knee injury: (A) mid-shaft femur fracture managed with interlocking nail and (B) mid-shaft tibia fracture managed with interlocking nail

**Figure 5:**
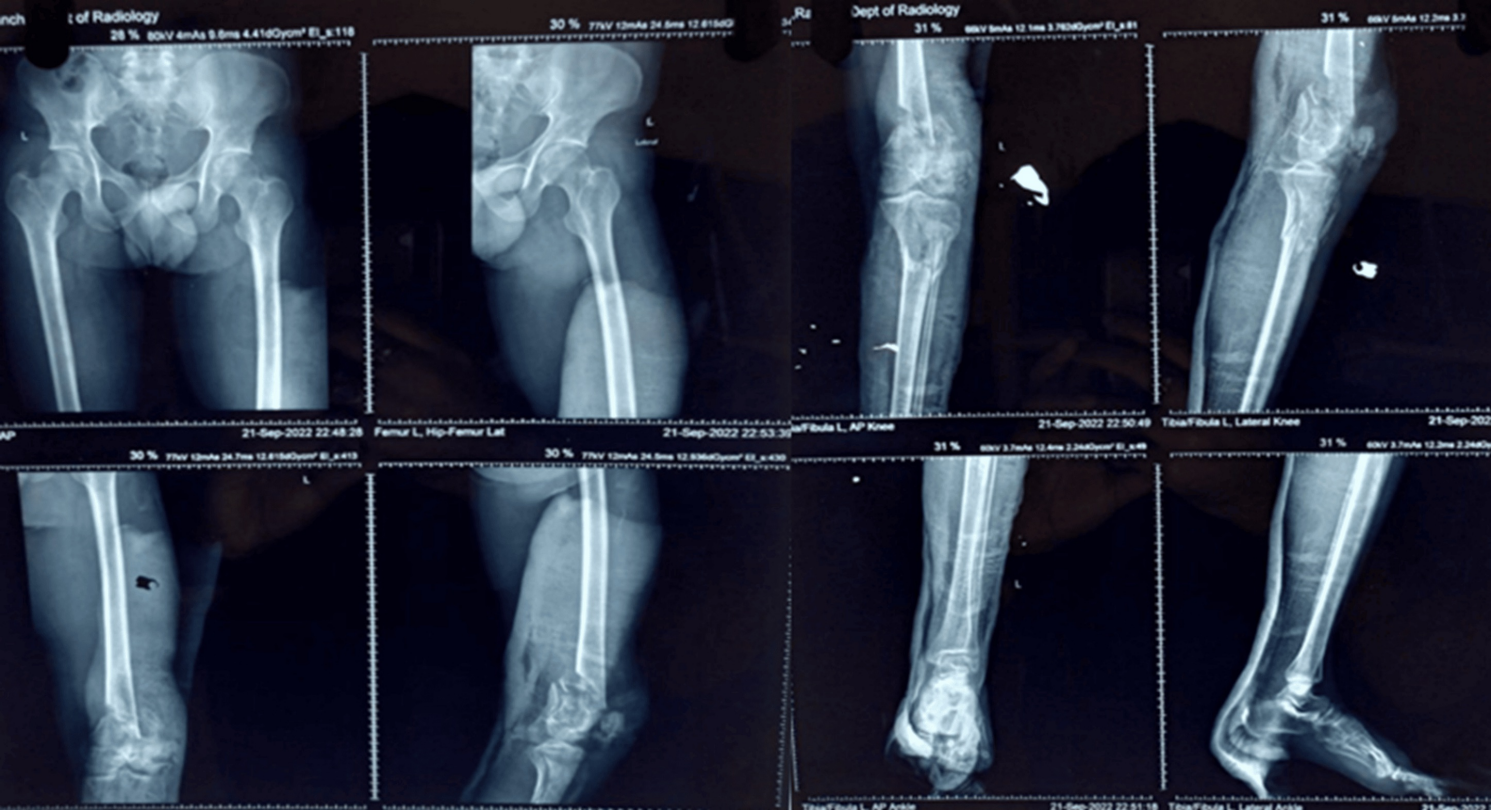
Preoperative radiograph showing a fracture of the distal end of the femur and the proximal tibia (both intra-articular) indicating a type IIc floating knee injury

**Figure 6:**
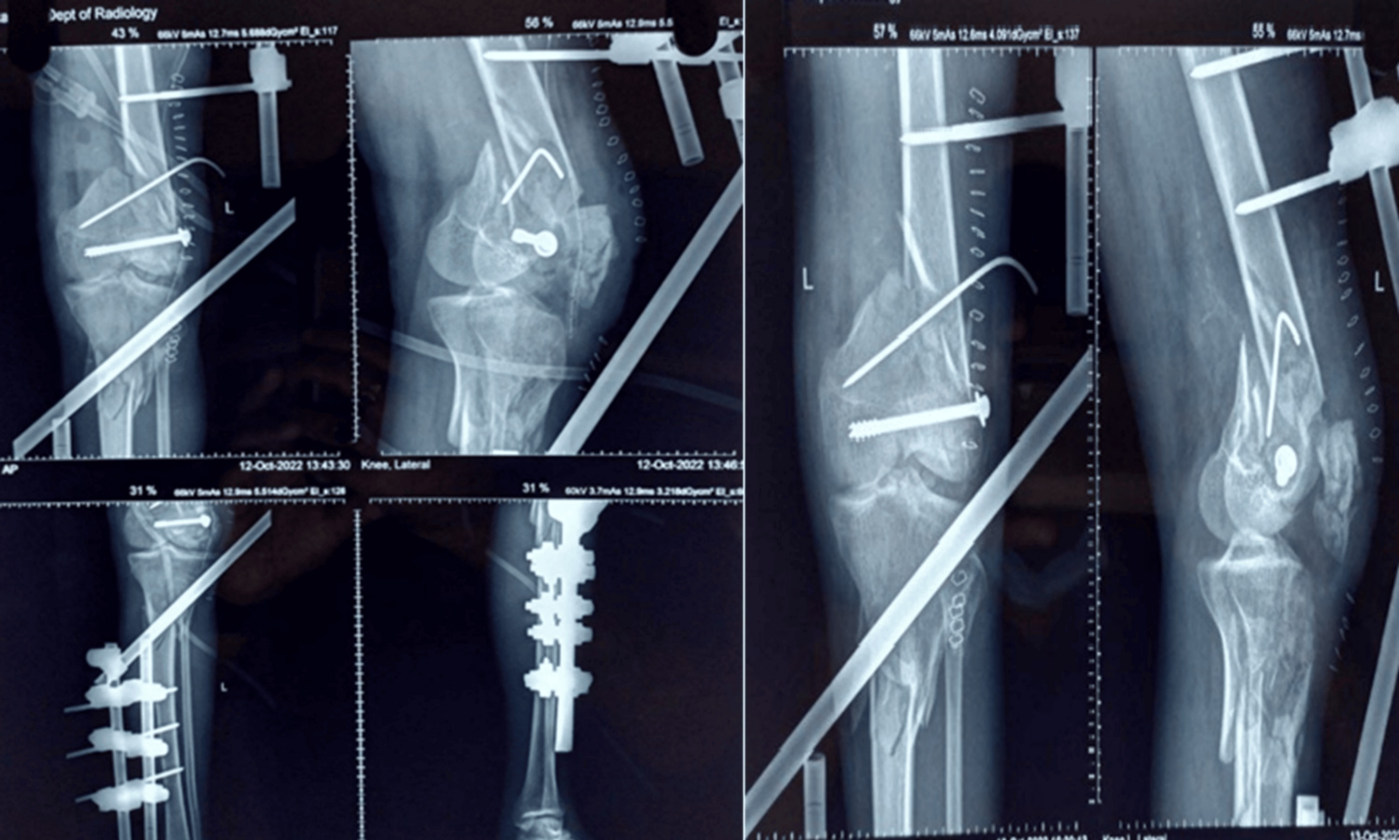
Postoperative radiograph showing a trans knee fixator with cortico-cancellous screw and K-wire in the distal femur in type IIc floating knee injury (open)

Hemodynamically stable patients with closed injuries were considered for immediate definitive fixation. In contrast, those with unstable conditions and open fractures were managed with temporary external fixation following principles of damage control orthopedics. Once the patient's condition stabilized, conversion to definitive internal fixation was performed as indicated (Figures 3-6).

Fractures of the tibia and femur were managed using intramedullary nails, periarticular locking compression plates, dynamic compression plates, or external fixators, with the choice of fixation method determined by factors such as the fracture's location and pattern, whether the injury was open or closed, the patient's overall condition, soft tissue involvement, and any associated injuries. Patients were followed up every four weeks until the fracture healed, and functional outcomes were assessed at the 1.5-year mark using the Karlstrom criteria (Table 1).

**Table 1:** The Karlstrom criteria for the functional assessment of floating knee injuries

Criterion	Excellent	Good	Acceptable	Poor
Subjective symptoms from the thigh or leg	None	Intermittent slight symptoms	More severe symptoms, impairing function	Considerable function impairment; pain at rest
Subjective symptoms from the knee or ankle joint	None	Same as above	Same as above	Same as above
Walking ability	Unimpaired	Same as above	Walking distance restricted	Uses a cane, crutch, or other support
Work and sports	Same as before the accident	Given up some sports: work the same as before the accident	Change to less strenuous activity	Permanent disability
Angulation, rotational deformity, or both	0	<10%	10-20°	>20°
Shortening	0	<1 cm	1-3 cm	>3 cm
Restricted joint mobility (hip, knee, ankle)	0	<10°at the ankle; <20°at the hip, knee, or both	10-20°at the ankle; 20-40°at the hip, knee, or both	>20°at the ankle; >40° at the hip, knee, or both

Statistical analysis

Sample Size Calculation

The sample size was calculated from the study of Rollo et al. [1]. The total sample size came out to be 40. The data collected were entered into Microsoft Excel data sheet (Microsoft Corporation, Redmond, Washington, United States), and data were checked carefully before entering. SPSS Statistics for Windows, Version 16.0 (Released 2007; SPSS Inc., Chicago, Illinois, United States) has been used for statistical analysis which adheres to the objectives of our study. Fisher's exact test and the chi-squared test were used to obtain the level of significance.

## Results

The mean age of the study participants was 37.6(±8.36) years. The minimum age was 21 years, while the maximum age was 54 years. The majority of patients (67.5%) were men, while women made up 32.5%. There were 24 closed cases and 16 open cases. Table 2 represents patients' details such as age, gender, affected limb, and type of injury.

**Table 2:** Patients' demographics in floating knee injury where data were presented as mean±SD or n (%)

Characteristics	Values
Age (in years)	37.6±8.36
Gender	
Male	27 (67.5%)
Female	13 (32.5%)
Affected limb	
Right limb	23 (57.5%)
Left limb	17 (42.5%)
Mode of injury	
Road traffic accidents	36 (90%)
Fall from height	4 (10%)
Type of injury	
Open	16 (40%)
Closed	24 (60%)

The p-value of 0.001 indicates a statistically significant association between the type of fracture (open vs. closed) and the functional outcome after 18 months. Table 3 shows the association of open/closed injury with functional outcomes after 18 months.

**Table 3:** Association of open/closed injury with functional outcome after 18 months where data were presented as n (%) Fisher's exact test was used to obtain the p-value. The p-value was considered significant at <0.05

Type of injury	Functional outcome (Karlstrom criteria) after 18 months				Fisher's exact test=23.093; df=3; p<0.001
	Excellent (n=16)	Good (n=13)	Acceptable (n=9)	Poor (n=2)	
Open (n=16)	2 (12.5%)	3 (23.1%)	9 (100%)	2 (100%)	
Closed (n=24)	14 (87.5%)	10 (76.9%)	0 (0%)	0 (0%)	

There is a strong and significant relationship between the type of injury (open or closed) and the radiological outcome of femur union with a p-value of <0.001. Table 4 depicts the association of open/closed fracture of the femur with radiological outcome.

**Table 4:** Association of open/closed fracture of the femur with radiological outcome where data were presented as n (%) The chi-squared test was used to obtain the p-value. The p-value was considered significant at <0.05

Type of fracture	Radiological outcome of the femur			
	Union at 9 months	Union at 12 months	Union at 18 months	p<0.001
Open (n=16)	1 (5.26%)	8 (57.14%)	7 (100%)	
Closed (n=24)	18 (94.74%)	6 (42.86%)	0 (0%)	

The p-value of <0.001 indicates a highly statistically significant association between the type of injury in open or closed and the radiological sign of tibia union at different time points. Table 5 represents the association of open/closed fracture of the tibia with radiological outcome.

**Table 5:** Association of open/closed fracture of the tibia with radiological outcome where data were presented as n (%) The chi-squared test was used to obtain the p-value. The p-value was considered significant at <0.05

Type of fracture	Radiological outcome of the tibia			
	Union at 9 months	Union at 12 months	Union at 18 months	p<0.001
Open (n=16)	2 (12.5%)	5 (31.3%)	9 (56.2%)	
Closed (n=24)	19 (79.2%)	5 (20.8%)	0 (0%)	

Floating knee injuries were associated with various complications in our study participants. Table 6 depicts complications in study participants.

**Table 6:** Distribution of complications in study subjects where data were presented as n (%)

Complication	Frequency
Common peroneal nerve injury	2 (5%)
Deep vein thrombosis	1 (2.5%)
Superficial infection	7 (17.5%)
Fat embolism	3 (7.5%)
Pin tract infection	4 (10%)
Delayed union of the femur	21 (52.5%)
Delayed union of the tibia	19 (47.5%)
Hip stiffness	12 (30%)
Knee stiffness	22 (55%)
Malunion of the femur	2 (5%)
Malunion of the tibia	8 (20%)
Shortening	2 (5%)
Knee instability	1 (2.5%)
Osteoarthritis	4 (10%)
Osteomyelitis	1 (2.5%)

## Discussion

This study was conducted to assess the management strategies, challenges, and clinico-radiological outcomes of the different types of floating knee injuries including their functional outcomes along with a comparison of the radiological outcomes.

In the present study, the mean age of patients was 37.6±8.36 years. Similarly, a study conducted by Kulkarni et al. found that the mean age of study participants was 34.34±12.8 years [6]. In addition, the gender distribution indicates a predominance of men, who comprise 67.5% of the sample, while women make up 32.5%. A study by Nouraei et al. reported that approximately 85.5% of their study participants were men [5].

The modes of injury in the study revealed that 92.5% of the floating knee injuries resulted from road traffic accidents (RTAs), while 7.5% were due to falls from height. A study by Rethnam et al. highlighted that RTAs are the most common cause of floating knee injuries, accounting for a significant majority of cases [8]. 

In our study, 60% of the floating knee injuries are classified as closed, while 40% are open injuries (with 30% open tibia fracture and 10% open femur fracture). According to the Fraser classification, there are 10 cases with type I fracture, 11 cases with type IIa, nine cases with type IIb, and 10 cases with type IIc. A study by Feron et al. also found that closed injuries were more prevalent than open injuries in floating knee cases [9].

The current study indicates that 42.5% of floating knee injuries affected the left limb, while 57.5% involved the right limb. This distribution is consistent with trends observed in the literature, suggesting a slight predominance of right-sided injuries [6,9,10].

The management of floating knee injuries showed promising functional outcomes at 18 months, based on the Karlstrom criteria. The largest group, 40% of patients, achieved excellent outcomes, reflecting effective treatment and rehabilitation. Another 32.5% had good outcomes, with high recovery levels and minor limitations. Acceptable outcomes were seen in 22.5% of cases, indicating satisfactory recovery with some restrictions. Only 5% of patients experienced poor outcomes, pointing to the need for further evaluation and refinement of treatment strategies for these individuals. The majority of cases (72.5%) in our study achieved either excellent or good functional outcomes. Around 87.5% of cases with excellent functional outcomes are closed injuries. Similarly, 76.9% of cases with good functional outcomes are closed injuries. The majority of type I injuries according to the Fraser classification have excellent functional outcomes, while acceptable to poor functional outcomes are seen mainly in type IIc injuries. This is consistent with literature indicating that with appropriate management, including timely surgical intervention and structured rehabilitation, patients with floating knee injuries can achieve high levels of functional recovery [10,11].

The radiological outcomes of femur union in the study reveal a substantial difference between open and closed injuries, supported by a highly significant p-value of <0.001. This underscores the robustness of the association between injury type and union time. Closed femur fractures tend to unite earlier, typically within nine months, whereas open fractures may require significantly longer, often showing delayed union patterns up to 12-18 months post-injury.

The radiological outcomes of tibia fracture union, comparing open and closed injuries, reveal a compelling association supported by a highly significant p-value. Delayed union in open tibia fractures is not uncommon and can significantly impact long-term outcomes, emphasizing the importance of meticulous management and monitoring to promote timely healing and reduce complications.

The distribution of complications provides valuable insights into the postoperative course for patients with floating knee injuries. The most common complication was a superficial infection (surgical site infection), occurring in seven cases (17.5%). Infection control is crucial in orthopedic surgery, especially in cases involving open fractures and the use of implants. Occurring in four cases (10.0%), pin tract infections are a known risk with external fixators. The high incidence of superficial and pin tract infections aligns with existing literature that underscores the challenges of infection control in orthopedic trauma surgery [12].

Delayed union of the femur occurred in 52.5% of cases. Delayed union of the tibia was noted in 47.5% of cases. Joint stiffness, particularly in the knee, can severely limit mobility and affect the patient's ability to perform daily activities. The high incidence of knee stiffness underscores the need for effective physiotherapy and rehabilitation protocols post-surgery. Strategies such as preoperative skin preparation, intraoperative sterility, and postoperative wound care are crucial. Prophylactic antibiotics can reduce the incidence of infections in open fractures.

The prospective nature of the study allows for the systematic collection of data, ensuring the temporal relationship between interventions and outcomes, which enhances the reliability of findings. The study tracks patients for an extended period, allowing for the evaluation of long-term outcomes, including fracture union and functional recovery. Conducting the study in Jharkhand highlights the challenges and solutions specific to the region, addressing a population that may be underrepresented in the literature. The limitations of the study were that it was a single-center study, and for generalization, a multicentric study is needed. 

## Conclusions

The study concluded that at 18 months, the majority of the patients showed good to excellent functional outcomes. Statistically significant associations were seen between the association of open or closed fractures and radiological outcomes. As the young working-age population is affected by high-energy traumas, proper classification of injuries with early evidence-based treatment is much needed to have a better quality of life without residual complications. Also, keeping a close watch on common early and late complications can improve the quality of life of the patients, and infection control for open fractures should be dealt with special attention.
